# Associations between patient factors and adverse events in the home care setting: a secondary data analysis of two canadian adverse event studies

**DOI:** 10.1186/s12913-017-2351-8

**Published:** 2017-06-12

**Authors:** Nancy A. Sears, Régis Blais, Michael Spinks, Michèle Paré, G. Ross Baker

**Affiliations:** 1 School of Baccalaureate Nursing, St. Lawrence College, Kingston, ON Canada; 2 Department of Health Administration, Public Health Research Institute, Université de Montréal, PO Box 6128, Station Centreville, Montreal, Québec H3C 3J7 Canada; 3 Data2Intel, Courtice, ON Canada; 4 Public Health Research Institute, Université de Montréal, Montreal, Québec, Canada; 5 Institute of Health Policy, Management and Evaluation, University of Toronto, Toronto, ON Canada

**Keywords:** Home care, Adverse events, Quality, Harm, Safety, Risk

## Abstract

**Background:**

Early identification of patients at who have a higher risk for the occurrence of harm can provide patient safety improvement opportunities. Patient factors contribute to adverse event occurrence. The study aim was to identify a single, parsimonious model of home care patient factors that, regardless of location and differences in home care program management and design factors, could provide a means of locating patients at higher and lower risk of harm.

**Methods:**

Split modeling using secondary analyses of data from two recent Canadian home care patient safety studies was undertaken. Patient factors from the Minimum Data Set Resident Assessment Instrument (RAI) for Home Care and diagnoses consistent with ICD-10 and RAI-Mental Health assessment were used. Continuous and categorical measures of factors were considered. Adverse events were defined using World Health Organization taxonomy and measured on a dichotomous *yes/no* scale. Patient factors significantly associated (Pearson’s Chi Square, *p* ≤ .05) with the occurrence of adverse events in both earlier studies were entered in forward selection regression analyses to locate factors predictive of adverse event occurrence.

**Results:**

Instrumental activities of daily living dependency and escalating co-morbidity counts are associated with patient vulnerability to adverse events.

**Conclusions:**

Instrumental activities of daily living dependency and burden of illness, both easily identifiable early in the episode of care, are significantly associated with the risk of adverse event occurrence, however there is regional variability in the relationships.

## Background

**Fig. 1:**
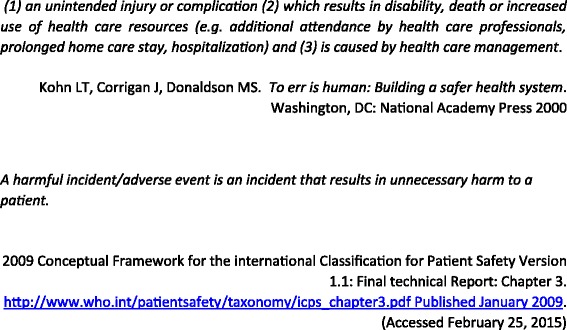
The 3 criteria definition of adverse event (AE)

Identifying circumstances where patients have increased risks for the occurrence of harm can be a means of promoting patient safety through improvements in health care processes and systems [1–4]. There are growing calls for improved safety in home care settings in a number of countries [5–7]. While there is an established, and growing, body of literature considering the identification and mitigation of risks for harm within broad population groups such as the elderly, and subpopulations including the elderly with functional limitations and/or environmental challenges such as isolation and/or limited financial resources [8–12], there is a limited body of literature examining factors associated with safety for home care patients. This latter body of evidence is based largely on the retrospective identification of the incidence and types of adverse events (AEs) [Fig. 1] that have occurred. [13–16] A mapping review of international literature considering safety in home care found few studies describing the prevalence and incidence of home care AEs, and strategies for reducing them. The review authors concluded that interventional research to evaluate home care strategies aimed at reducing the risks of patient harm will advance only when analysis of the location and description of the circumstances of AEs within home care improves [17].

Vincent’s accident causation model identifies multiple factors that can cause patient harm thus providing a conceptual framework for studying patient safety [18]. Patient factors are one of the factors that produce conditions that contribute to AEs; thus studies that locate and measure AEs in home care may inform efforts to reduce their incidence, and in extension, the harm that home care patients encounter as a result of AEs.

Two recent Canadian studies that used almost identical sampling and data collection methodology, reported AE incidence rates of 13.2% (95% CI 10.4%–16.6%) amongst Ontario home care patients [19] and 10.1% (95% CI 8.4%–11.8%) across an aggregated sample from Manitoba, Quebec and Nova Scotia [20]. The more common types of AEs found in both studies were falls with injuries (Ontario: 24.6% [15 of 61] of AEs; Manitoba, Quebec, Nova Scotia: 17.2% [16 of 93] of AEs), medication related AEs (Ontario 16.4% [10/61]; Manitoba, Quebec, Nova Scotia: 6.5% [6/93]); infections (Ontario 8.2% [5/61]; Manitoba, Quebec, Nova Scotia: 18.3% [17/93]; and pressure ulcers (Ontario 11.5% [7/61]; Manitoba, Quebec, Nova Scotia: 6.5% [6/93]). Deaths resulting from AEs were found in both studies (Ontario: 6 deaths across 61 AEs; Manitoba, Quebec, Nova Scotia: 7 deaths across 93 AEs). Both studies also identified multiple factors that individually were significantly associated (χ^2^, *p* ≤ .05) with the occurrence of an AE. Detailed tables reporting the factors found to be significantly associated with AEs are reported in the referenced papers. Common significant factors across the two analyses were dependency for several instrumental activities of independent living (IADLs) and several diagnoses such as cardiac or vascular diseases/conditions and neurological disorders. The identification of factors that can be assessed easily and early in the provision of home care represents a significant opportunity to avoid AEs and thereby improve patient safety through enhanced health care management such as improvements in case management as well as clinical and supportive care processes [19, 20].

In individual reports of the two studies (Ontario and Manitoba, Quebec and Nova Scotia) regression analyses provided predictive modelling that was able to identify home care patients at higher or lower risk for experiencing an AE [19, 20]. There were differences between the models produced within the two studies. We were interested in determining if data from both of these two Canadian studies could be used to develop a single, parsimonious model of factors within the home care patient population that, regardless of location and differences in home care program management and design factors, could help identify patients with higher and lower risk of experiencing harm. To do this we applied the model from the second study (Quebec, Manitoba, Nova Scotia) to the Ontario data. This split modeling approach across the two studies provides a method of validation across provincial programs.

## Methods

This was a secondary analysis of the data from two retrospective chart review-based studies and is compliant with the STROBE Statement for reporting observational studies in epidemiology. SPSS version 23 was used for statistical analysis; the level of significance used was .05.

The first study used a stratified sample of 430 randomly selected patients from a sampling frame of 7467 (α 0.05, CI >99%, ER 2.91) across three contiguous home care jurisdictions in Ontario, Canada. The strata sample weights represented between 16 and 19% of home care patients in each of the three subregions and the sample was treated as an approximation of a simple random sample. All patients had received nursing services and been discharged between April 1st 2004 and March 31st 2005. No other exclusionary criteria were applied to the sample; no sample substitutions were made [19]. The second study used a stratified random sample of 1200 patients (α 0.05, CI >95%, ER 1.84) who had been discharged between April 1st 2009 and March 31st 2010 from home care programs in the Winnipeg, Manitoba region, in 10 local health territories in Quebec and in the Halifax and Sydney regions of Nova Scotia. Again, no other exclusionary criteria were applied to the sample [20]. Home care programs for all four provinces admit patients of all ages (birth to over 100 years of age), needing acute or longer term health care services for purposes of curative or recovery care, rehabilitation, maintenance care for significant chronic conditions, and end-of-life care. In the absence of home care, most patients would require admission to hospitals or other health care institutions. As a retrospective study with secondary analyses of anonymized data and no risk to participants, no consents were required. Ethics approvals for the original studies were obtained from the Research Ethics Boards for the University of Toronto, the University of Manitoba, the Winnipeg Regional Health Authority, the University of Montreal, Dalhousie University, the Victorian Order of Nurses and each participating Centre of Health and Social Services in Quebec that required it (five out of ten). These approvals included the circumstances within which this secondary analysis was conducted.

In both studies, AEs were identified using the two-phase retrospective chart analysis methods previously used in hospital adverse event studies [3, 21–24]. This method has moderate to good reliability [25–29]. Patients’ case management files that contained clinical summaries were included as data sources. The Minimum Data Set Resident Assessment Instrument for Home Care (MDS RAI-HC) was generally used by the home care programs to document standardized patient assessments and, along with the narrative patient charts, provided the source of all data in the studies. Data elements from MDS RAI-HC describe patient functional, cognitive, mood, behavioural, vision, communication, nutrition and symptom domains, and are acceptable for research studies [30]. Diagnoses used to determine morbidities were consistent with ICD-10 and RAI-Mental Health assessment items. No differences in patient data coding practices between provinces were noted.

Within both studies, variables significantly associated (*p* ≤ .05) with the occurrence of AEs were identified using Pearson’s Chi Square. These variables were classified into blocks that contained similar content (i.e., patient demographics, admission/discharge factors, clinical history, functional status and co-morbid diagnoses). Given that the outcome of interest (presence of an AE) was measured on a dichotomous *yes/no* scale, and the samples were considered to be simple random distributions, multiple logistic regression analyses were used for determination of significant predictors. Within the analyses for each study, multiple demographic and patient clinical factors were tested and each variable significantly associated (Pearson’s Chi Square, *p* ≤ .05) with the occurrence of AEs was included and had the same chance of being included into the regression model of potential predictor factors. For consistency, variables for all provinces were selected using forward stepwise elimination. The studies and these analyses are more comprehensively described in the two earlier papers [19, 20].

Comparison of the results of the Manitoba, Quebec and Nova Scotia study with the Ontario study revealed that lack of independence for varying IADLs (generally recorded across all provinces using the RAI-HC data instrument) and diagnoses/morbidity factors were strongly predictive of AE occurrence in both studies. However, while the data for these two factors were comparable across the two studies, for each home care patient in the three province study an IADL dependency score was constructed as the mean of values across 7 IADLs (meal preparation, housework, financial transactions, medication administration, telephone use, shopping, transportation), where values are: 1 = independent, 2 = completes with difficulty, 3 = requires assistance, 4 = dependent. In addition, a simple count of co-morbidities for each patient was calculated. The maximum number of comorbidities found for any single patient was 15. Similar analysis of data aggregated in this fashion had not occurred in the original analysis for the Ontario study.

To create a similar analysis, the methods for aggregating IADL dependency scores and co-morbidities counts were recreated in the Ontario study data and regression analysis was used to determine if these factors are predictive of AE occurrence.

To determine if there is an upper or lower threshold at which IADL dependency or the number of co-morbidities indicates a greater or lesser odds for the occurrence of AEs, regression analysis was also undertaken with IADL scores and the number of co-morbidities each grouped into three categories (IADLs: 0 = independent; 1 = completes with difficulty or requires assistance; 2 = dependent. Number of co-morbidities: 0–2 co-morbidities, 3–6 co-morbidities, 7+ co-morbidities).

Modeling results from the three province study were also tested at the single province level to determine if the results were applicable at this smaller level or if there was small area variation that would affect application of the model.

## Results

Logistic modeling for AEs was carried out for each of the four provinces. In the first series of analyses (Table 1), where IADL dependency scores and number of co-morbidities were measured as continuous variables, IADL dependency scores were significantly related (*p* ≤ .05) to AEs (more dependent patients had higher odds of experiencing AEs) within the home care samples from Nova Scotia, Manitoba and Ontario, but not Quebec. Co-morbidity counts were significantly related (*p* =.005) to AEs (sicker patients are had higher odds of experiencing AEs) for Quebec and Nova Scotia, but not for Manitoba or Ontario.

**Table 1:** Results of Logistic Regression Models ^a^: Continuous IADL Score^b^ and Number of Diagnoses as Predictors of Adverse Events in Four Canadian Provinces

	p	O.R.	95% CI for O.R.	
			Lower	Upper
Quebec (*n* = 602)				
IADL Dependency and Number of Diagnosis				
Constant	0.000	0.027		
IADL dependency	0.063			
Mean # diagnoses	0.005	1.213	1.060	1.388
Nova Scotia (*n* = 302)				
IADL Dependency and Number of Diagnosis				
Constant	0.000	0.016		
IADL dependency	0.040	1.576	1.020	2.434
Mean # diagnoses	0.001	1.265	1.099	1.456
Manitoba (*n* = 296)				
IADL Dependency and Number of Diagnosis				
Constant	0.000	0.010		
IADL dependency	0.033	1.902	1.054	3.432
Mean # diagnoses	0.102			
Ontario (*n* = 430)				
IADL Dependency and Number of Diagnosis				
Constant	0.000	0.052		
IADL dependency	0.000	2.832	1.790	4.481
Mean # diagnoses	0.065			

^a^Forward stepwise regression used. n.s. signifies factor was not significant

^b^IADL dependency score is the mean of values across 7 IADLs (meal preparation, housework, financial transactions, medication administration, telephone use, shopping, transportation), where values are: 1 = independent, 2 = completes with difficulty, 3 = requires assistance, 4 = dependent

Results of the second series of analyses (Table 2), where IADL dependency and number of co-morbidities were categorized, showed that an IADL dependency score greater than 1 for Quebec, and scores between 0.5 and 1 and greater than 1 for Nova Scotia and Ontario were significantly (*p* ≤ .05) related to AEs. Categorized IADL dependency scores were not significantly (*p* ≤ .05) related to AEs for Manitoba. Co-morbidity counts, when measured as a categorized variable, were not significantly (*p* ≤ .05) related to AEs in Quebec. Having seven or more co-morbidities indicated higher odds for the occurrence of AEs in Nova Scotia (*p* = .004). Categorized co-morbidity counts were not significantly (*p* ≤ .05) related to AEs for Ontario or Manitoba.

**Table 2:** Results of Logistic Regression Models^a^: Categorical IADL Score^b^ and Number of Diagnoses as Predictors of Adverse Events in Four Canadian Provinces

	p	O.R.	95% CI for O.R.	
			Lower	Upper
Quebec (*n* = 602)				
IADL Dependency and Number of Diagnosis				
Constant	0.000	0.037		
IADL dependency	0.006			
IADL dependency (1)	0.849	0.878	0.231	3.344
IADL dependency (2)	0.014	3.522	1.293	9.590
Mean # diagnoses	0.156			
Mean # diagnoses (1)	0.332			
Mean # diagnoses (2)	0.060			
Nova Scotia (*n* = 302)				
IADL Dependency and Number of Diagnosis				
Constant	0.000	0.025		
IADL dependency	0.046			
IADL dependency (1)	0.040	3.309	1.057	10.354
IADL dependency (2)	0.014	4.407	1.353	14.351
Mean # diagnoses	0.016			
Mean # diagnoses (1)	0.126	2.052	0.817	5.149
Mean # diagnoses (2)	0.004	5.154	1.684	15.771
Manitoba (*n* = 296)				
IADL Dependency and Number of Diagnosis				
Constant	0.000	0.058		
IADL dependency	0.176			
IADL dependency (1)	0.745			
IADL dependency (2)	0.085			
Mean # diagnoses	0.167			
Mean # diagnoses (1)	0.456			
Mean # diagnoses (2)	0.428			
Ontario (*n* = 430)				
IADL Dependency and Number of Diagnosis				
Constant	0.000	0.306		
IADL dependency	0.000			
IADL dependency (1)	0.000	0.197	0.088	0.439
IADL dependency (2)	0.001	0.287	0.136	0.604
Mean # diagnoses	0.112			
Mean # diagnoses (1)	0.033			
Mean # diagnoses (2)	0.102			

^a^Forward stepwise regression used. n.s. signifies factor was not significant

^b^IADL dependency score is the mean of values across 7 IADLs (meal preparation, housework, financial transactions, medication administration, telephone use, shopping, transportation), where values are: 0 = independent, 1 = completes with difficulty or requires assistance, 2 = dependent

## Discussion

Home care patients with higher IADL dependency and/or a higher number of diagnoses were found to have higher odds for experiencing an AE in all four Canadian provinces. The relationships between AE occurrence and these two factors, either singularly or jointly, varied by province and by whether these variables were considered as continuous or categorical measures. Home care programs, and indeed most healthcare in Canada fall under provincial jurisdiction. Legislation, regulations and policies defining the parameters within which patients are eligible for publically funded services, vary across provincial borders. Despite similarities in the purpose of home care across Canada, programs are organized on a provincial basis with differences in approaches to case management, the range of professional services provided, the mix of public and private sector workers, the levels of funding and the eligibility of clients and clients’ obligation to contribute to the costs of care [31, 32]. Thus it is possible that the variation in the odds of AE occurrence associated with IADL dependency and comorbidity counts resulted from differences in the characteristics of the home care programs and variations in healthcare and other services provided to home care patients in these provinces.

These findings advance the understanding of patient safety within the home care population by establishing that there are similar, but not identical, conditions related to higher or lower propensity for patients to experience unintended harm related to the provision of health care services in different jurisdictions. While these conditions may be similar within a single province, there is sufficient variance between provinces that modelling of the factors that help identify patients with higher and lower risk of experiencing AEs may need to be conducted for each province.

Previous research has shown that more vulnerable home care patients are more susceptible to experiencing an AE [20]. Various interventions to enhance safety and functioning within populations such as the elderly, with or without disability, living independently or with community supports, have been investigated [8, 11]. For example, in a randomized clinical trial, Szanton et al. demonstrated that an interdisciplinary team of a nurse, occupational therapist and handyman could intervene to reduce harm in a cohort of lower income older adults with disabilities [12]. Reviews of specific risks in elderly populations, such as falls, have also shown that some interventions can reduce the risks of falls [27]. Effective interventions rely on assessments that identify those at risk. Thus this study contributes to the evidence supporting the development and use of screening tools for populations, such as those receiving home care services. Risk assessment models that help identify and locate patients at higher or lower odds of experiencing AEs can guide the deployment of interventions to reduce these risks. Based on the analyses presented here, IADL dependency and diagnostic factors such as the numbers of co-morbidities, both of which are identifiable early in the episode of care, could be used to identify patients for whom enhanced levels of case management and interprofessional vigilance may be beneficial. Such vigilance could help in identifying, mitigating and responding to preventable AEs.

Risk assessment models and tools that provide home care administrators, case managers and clinicians with the means to identify patients or patient groups that have different propensities for experiencing an AE, can improve patient safety. The incorporation of screening for factors such as IADL dependency and/or co-morbidity count that reflect the likelihood of AEs for home care patients into the standard assessment of patients at the time of admission and routinely throughout the home care stay, could enable managers and clinicians to target enhanced care management and improvements in clinical and supportive care processes. While a single, parsimonious model of factors within the home care patient population that locates patients with higher and lower risk of experiencing harm remains elusive, the results indicate that increased IADL dependency alone or in combination with increasing numbers of comorbidities should be taken into consideration in managing care.

The analysis in this paper is based on data from four Canadian provinces. The findings may not be relevant to patients in other provinces, or other settings. A larger data set that includes information on patient characteristics and program structures and services would help to refine the results reported here.

Advancements in the integration of integrated care across home and hospital settings also presents new patient safety risks. Patients with multiple co-morbid conditions are increasingly attended by an array of organizations (e.g., hospitals, clinics, medical practices, pharmacies, medical supply companies, home care programs, healthcare personnel agencies, community support agencies, etc.) and personnel (e.g., hospital staff, primary care physicians and nurses, home care agency nurses, therapists and supporting staff, community pharmacists, etc.) whose work is often insufficiently coordinated. Emerging safety risks may be expressed through increasing odds for AEs within the home care patient population [33]. Future AE studies that examine not only patient factors, but also home care program factors, and their relationships to AE occurrence are needed to advance the understanding and prevention of home care patients’ vulnerability to AEs. In addition, future studies should investigate the effects that risk-mitigating interventions have in enhancing safety within the home care population. Due to the possibility of variance across patient, environmental and home care program factors, matched subject groups within randomized clinical trials would provide insight into the effects of single and grouped sets of interventions while reducing the chances of unidentified and uncontrolled factors skewing the results. Epidemiological, retrospective studies should include larger samples, across different home care programs. Future prospective and retrospective studies need to examine behavioural and environmental factors that influence the vulnerability of those in home care to AEs, as well as to expand evidence of strategies and interventions that contribute to safety for seniors living independently.

## Conclusions

While it is possible and important to use evidence to identify patients with higher and lower risk of experiencing unintended harm, and to attempt to mitigate the potential for such harm, this knowledge may need to be developed at regional rather than global levels. Home care administrators, case managers, and clinicians need to acknowledge the incidence of adverse events in home care. Greater vigilance for all patients coupled with targeted interventions for patients assessed as higher risks may contribute to limiting the unintended harm experienced by vulnerable patients in the home setting. The results of this study suggest that patients with higher IADL dependence and higher number of co-morbidities should be identified, both at the time of admission to the home care program, and at frequent and regular intervals. Assessment tools, including both existing instruments such as the Resident Assessment Instrument for Home Care (RAI-HC) [34] and new tools could be useful for the identification of high-risk patients. Those patients with greater vulnerability could then be offered additional care oversight and other interventions to mitigate risk.

## Abbreviations

AE: Adverse event
IADL: Instrumental activities of daily living
MDS RAI-HC: Minimum data set resident assessment instrument for home care

## References

[1] Brennan TA, Leape LL, Laird NM, Hebert L, Localio AR, Lawthers AG (1991). Incidence of adverse events and negligence in hospitalized patients. Results of the Harvard Medical Practice Study I. N Engl J Med.

[2] Schiøler T, Lipczak H, Pedersen BL, Mogensen TS, Beck KB, Stockmarr A, Svenning AR, Frølich A.Incidence of adverse events in hospitals. A retrospective study of medical records. Ugeskr Laeger. 2001;163(39):5370–8. 11590953

[3] Wilson RM, Runciman WB, Gibberd RW, Harrison BT, Newby L, Hamilton JD (1995). The quality in Australia health care study. Med J Aust.

[4] Thomas EJ, Studdert DM, Burstin HR, Orav EJ, Zeena T, Williams EF (2000). Incidence and types of adverse events and negligent care in Utah and Colorado. Med Care.

[5] Joint Commission (USA). National Patient Safety Goals: Home Care Accreditation Program. 2016. https://www.jointcommission.org/assets/1/6/2016_NPSG_OME.pdf. Accessed 19 May 2016.

[6] Canadian Patient Safety Institute: Patient safety forward with four 2013–2018. http://www.patientsafetyinstitute.ca/en/about/patientsafetyforwardwith4/Pages/default.aspx. Accessed 19 May 2016.

[7] Canadian Patient Safety Institute (2014). Integrated patient safety action plan.

[8] Gitlin LN, Winter L, Dennis MP, Corcoran M, Schinfeld S, Hauck WW (2006). A randomized trial of a multicomponent home intervention to reduce functional difficulties in older adults. J Am Geriatr Soc.

[9] Beswick AD, Rees K, Dieppe P, Ayis S, Gooberman-Hill R, Horwwod J (2008). Complex interventions to improve physical function and maintain independent living in elderly people: a systematic review and metaanalysis. Lancet.

[10] Daniels R, Metzelthin SF, van Rossum E, de Witte LP, van den Heuvel W (2010). Interventions to prevent disability in frail community-dwelling older persons: an overview. Eur J Ageing.

[11] Gillespie LD. Interventions for preventing falls in older people living in the community. Cochrane Database Syst Rev. 2012;9. doi:10.1002/14651858.CD007146.pub3 . 10.1002/14651858.CD007146.pub3 PMC8095069 22972103

[12] Szanton SL, Wolff JW, Leff B, Thorpe RJ, Tanner EK, Boyd C (2014). CAPABLE trial: a randomized controlled trial of nurse, occupational therapist and handyman to reduce disability among older adults: Rationale and design. Contemp Clin Trials.

[13] Johnson KG (2004). Adverse events among Winnipeg home care clients. Healthc Q.

[14] Madigan EA, Tullai-McGuinness (2004). An examination of the most frequent adverse events in home care agencies. Home Healthc Nurse.

[15] Meredith S, Feldman PH, Frey D, Hall K, Brown NJ, Ray WA (2001). Possible medication errors in home healthcare patients. J Am Geriatr Soc.

[16] Ellenbeker CH, Frazier SC, Verney S (2004). Nurses’ observations and experiences of problems and adverse effects of medication management in home care. Geriatr Nurs.

[17] Harrison M, Keeping-Burke L, Godfrey C, Ross-White A, McVeety J, Donaldson C, et al. Safety in home care: a mapping review of the international literature. Int J Evid Based Healthc. 2013;11:148–60.

[18] Vincent C (2003). Understanding and responding to adverse events. N Engl J Med.

[19] Sears N, Baker GR, Barnsley J, Shortt S (2013). The incidence of adverse events among home care patients. Int J Qual Health Care.

[20] Blais R, Sears NA, Doran D, Baker GR, Macdonald M, Mitchell L, et al. Assessing adverse events among home care clients in three Canadian provinces using chart review. BMJ Qual Saf. 2013;22:989–97. doi:10.1136/bmjqs-2013-002039. 10.1136/bmjqs-2013-002039 PMC3888609 23828878

[21] Vincent C, Neale G, Woloshynowych M (2001). Adverse events in British hospitals: preliminary retrospective record review. Br Med J.

[22] Baker GR, Norton RG, Flintoft V (2004). The Canadian adverse events study: the incidence of adverse events among hospital patients in Canada. CMAJ.

[23] Leape LL, Brennan TA, Laird N (1991). The nature of adverse events in hospitalized patients. Results of the Harvard Medical Practice Study II. N Engl J Med.

[24] Davis P, Lay-Yee R, Schug S, Briant R, Scott A, Johnson S, et al. Adverse event regional feasibility study: indicative findings. N Z Med J. 2001;114(1131):203–5. 11421433

[25] Walshe K (2000). Adverse events in health care: issues in measurement. Qual Health Care.

[26] Thomas EJ, Studdert DM, Brennan TA (2002). The reliability of medical record review for estimating adverse events. Ann Intern Med.

[27] Hripcsak G, Bakken S, Stetson PD (2003). Mining complex clinical data for patient safety research: a framework for event discovery. J Biomed Inform.

[28] Michel P, Quenon JL, de Sarasqueta AM (2004). Comparison of three methods for estimating rates of adverse events and rates of preventable adverse events in acute care hospitals. Br Med J.

[29] Lilford RJ, Mohammed MA, Braunholtz D, Hofer TP (2003). The measurement of active errors: methodological issues. Qual Saf Health Care.

[30] Sangl J, Saliba D, Gifford DR, Hittle DF (2005). Challenges in measuring nursing home and home health quality: lessons from the first national health care quality report. Med Care.

[31] Dumont-Lemasson M, DonovanC, Wylie M (1999). Provincial and territorial home care programs: a synthesis for Canada.

[32] Canadian Home Care Association (2013). Portraits of Home Care in Canada.

[33] Doran D, Blais R, Baker GR, Harrison MB, Lang A, Macdonald M, et al. The safety at home study: an evidence base for policy and practice change. Healthc Q. 2014;17(3):42–7. 10.12927/hcq.2014.24019 25591609

[34] Landi F, Tua E, Onder G, Carrara B, Sgadari A, Rinaldi C, et al. Minimum data set for home care: a valid instrument to assess frail older people living in the community. Med Care. 2000;38:1184–90. 10.1097/00005650-200012000-00005 11186297

